# A Rare Case of Human Herpesvirus 6 Meningitis in an Immunocompetent Asian Male Presented With a Severe Intractable Headache

**DOI:** 10.7759/cureus.15331

**Published:** 2021-05-30

**Authors:** Roshniben Patel, Akhila Mohan, Kishor Pokharel, Maria Pardi

**Affiliations:** 1 Internal Medicine, Saint Agnes Hospital, Baltimore, USA

**Keywords:** meningitis, meningoencephalitis, encephalitis, central nervous system infection, human herpes virus

## Abstract

Human herpesvirus 6 (HHV-6) manifesting as a central nervous system (CNS) infection (especially meningoencephalitis) is reported as a primary infection in children and from reactivation in immunocompromised patients; however, it has rarely been reported in immunocompetent adults. Latent infections of the CNS can cause a myriad of clinical presentations ranging from a benign, febrile, self-resolving illness to limbic encephalitis, temporal lobe seizures, and neuropsychiatric symptoms such as behavioral disturbances and psychosis. No standard diagnostic criteria or management guidelines exist for this condition. Possible neuroimaging findings include abnormalities in the medial temporal lobe involving the hippocampus and amygdala. We hereby present a case of HHV-6 meningitis in a 48-year-old immunocompetent male presenting without encephalopathic symptoms and normal neuroimaging findings.

## Introduction

Human herpesvirus 6 (HHV-6) is a member of the Herpesviridae family, a ubiquitous virus and the causal agent of roseola infantum that causes a mild and self-limiting illness in children within the first three years of life [1]. In 1986, HHV-6 was first isolated from peripheral blood mononuclear cells (PBMC) of patients with HIV and lymphoproliferative disorders, and it was further subclassified into variants A and B in 1993 [1,2]. HHV-6B is responsible for the majority of primary infections and cases of reactivation. Being from the Herpesviridae family, HHV-6 has a life-long latency in PBMC, salivary glands, and brain tissue following the primary infection due to viral genome integration into the host cell’s DNA [2,3]. In adults, primary infection is relatively uncommon. Reactivation or latent infection is mainly found in immunocompromised patients, particularly in HIV positive and hematopoietic stem cell or solid organ transplant patients in whom it can cause pneumonitis, hepatitis, and meningoencephalitis [1,4]. In contrast, immunocompetent adults very rarely have central nervous system (CNS) infection; nonetheless, there have been reported cases of severe CNS HHV-6 infections in immunocompetent adults presenting with deadly forms of the disease including status epilepticus, multiorgan failure, and meningoencephalitis [5]. Atypical presentations can lead to delays in diagnosis and treatment. We hereby present a rare case of HHV-6 meningitis in a 48-year-old, previously healthy, immunocompetent male patient presenting with severe intractable headache.

## Case presentation

A 48-year-old Asian male with no significant medical history presented complaining of a diffuse and persistent severe headache for 10 days. Associated symptoms included nausea, vomiting, poor appetite, and chills. He denied fever, neck pain, changes in vision, photophobia, focal weakness, and/or recent travel. Prior to this presentation, he had been seen in the ED and urgent care for these symptoms where he was diagnosed with tension-type headache and was prescribed analgesic, antiemetic, and abortive migraine medications such as triptan. He denied a past history of possible exposure to a person infected with HHV-6. The headache persisted despite these measures. On physical examination, he had a temperature of 39°C, a heart rate of 72 beats/minute, and a blood pressure of 79/50 mmHg. Neurological examination was normal including negative meningeal signs. Initial laboratory findings revealed the following: normal white blood cells 6.1 K/uL (normal range: 4-11 K/uL), low lymphocytes 15.7% (normal range: 20-45%), low hemoglobin 11 g/dl (normal range: 13-17 g/dl), low platelet count 144 K/uL (normal range: 150-400 K/uL); HIV, urine toxicology, and blood culture were negative. Despite the absence of meningeal signs, his fever, severe persistent headache and other symptoms were highly concerning for possible meningitis. A lumbar puncture followed and he was treated with intravenous fluids and empiric antibiotic and antiviral agents. Opening pressure was not measured. Cerebrospinal fluid (CSF) analysis showed elevated white blood cells (WBC) 1056 cells/uL (normal range: 0-10/uL) with 96% mononuclear cells, low glucose 39 mg/dL (normal range: 40-70 mg/dL), elevated total protein 268 mg/dL (normal range: 15-45 mg/dL), negative Gram stain; CSF BioFire was positive for HHV-6. HHV-6 deoxyribonucleic acid (DNA) polymerase chain reaction (PCR) viral load was not ordered on initial admission. After consultation with infectious disease, treatment with ganciclovir was started and all other antimicrobial agents were discontinued. 

**Figure 1:**
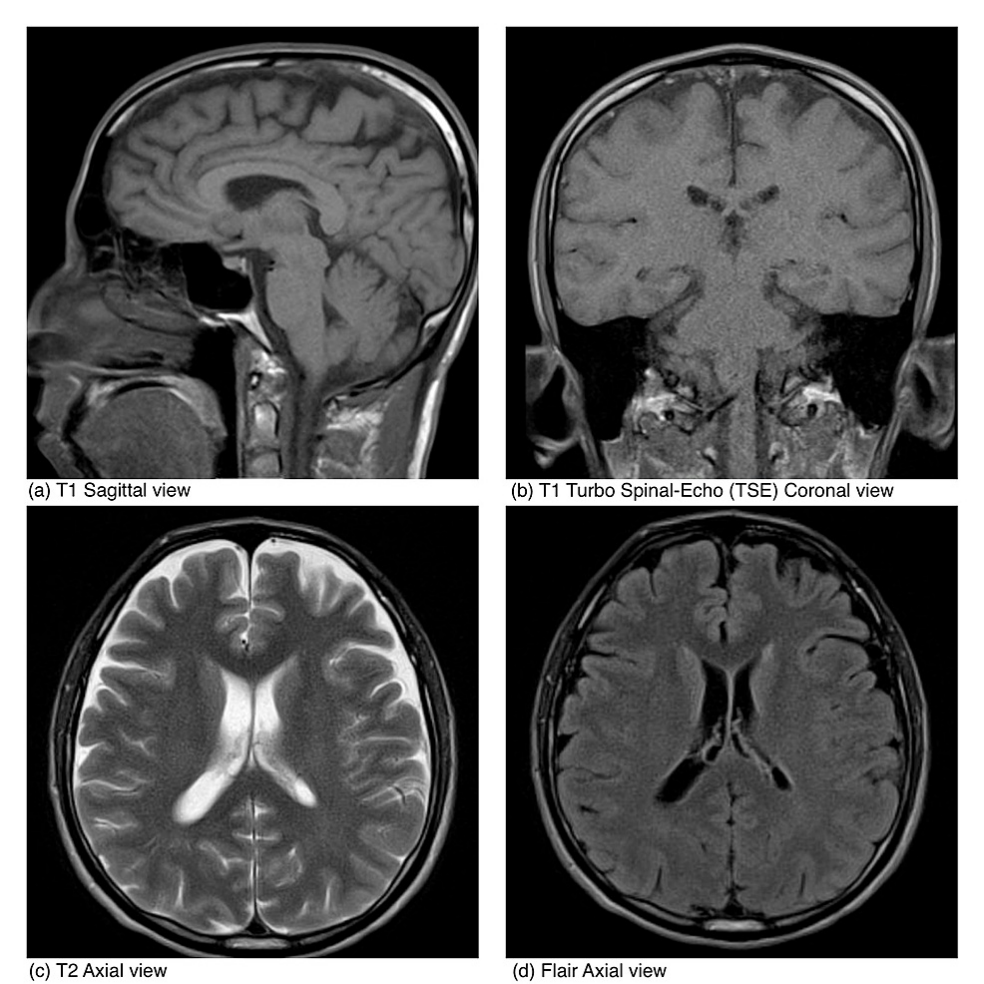
MRI without contrast.

Follow-up laboratory results within 24 hours were as follows: CSF culture remained negative, HHV-6 DNA PCR viral load <1000 copies/ mL, CSF was negative for other viruses including Herpes simplex virus 1 and 2, enterovirus, cytomegalovirus, and varicella-zoster virus. Serum immunoglobulin levels were IgG 617 mg/dl (normal range: 700-1600 mg/dL), IgA 128 mg/dl (normal range: 70-400 mg/dL), and IgM 72 mg/dl (normal range: 40-230 mg/dL). Brain MRI without intravenous contrast was normal (Figure 1).

Over the next few days, the patient’s headache gradually improved and completely resolved before discharge. He was discharged on oral valganciclovir for a total of 21-day course therapy. The patient has been doing well since discharge from the hospital; he completed the course of valganciclovir therapy and has no residual symptoms after one month of completion of therapy. HHV-6 DNA PCR viral load was not done during the follow-up visit.

## Discussion

HHV-6 is a lymphotropic and neurotropic virus that generally causes a mild, self-limited syndrome during childhood [1,3]. Almost all adults demonstrate antibodies to this virus. In adults, HHV-6 typically only causes illnesses in the setting of immunosuppression from conditions like HIV, transplantation, and reactivation of latent infection [1,3]. Among transplant recipients, it is commonly seen in unrelated cord blood cell transplantation and repeated hematopoietic stem cell transplantation [4].

According to the literature, the median age of presentation is 29 years (range between 18 and 85 years of age) with an interquartile range of 34.5 [3]. Males are predominantly affected around 33% more as compared to females [3,4]. HHV-6 infection can present with a wide variety of symptoms ranging from benign febrile exanthems to seizures and meningoencephalitis [6]. Even though there are case reports of complicated clinical presentations such as meningitis in immunocompetent individuals, these are very rare [5]. Although infrequent, it can also present with memory impairment, focal neurological deficits, hyponatremia, nystagmus, dysesthesia, and hemophagocytosis [4]. HHV-6 meningitis does not appear to have a typical presentation. Studies are lacking regarding the incidence and prevalence of this condition. There does not seem to be any seasonal, geographic, and/or ethnic predilection [6]. The risk factors and pathogenesis are yet to be completely elucidated but possible risk factors include male gender, certain genetic disorders, human leukocyte antigen (HLA) constellations, and immune tolerance to HHV-6 replication [4]. 

CSF analysis may demonstrate elevated protein and lymphocytic pleocytosis (mainly monocytic) [1,3]. CSF PCR for HHV-6 has more than 95% sensitivity, a positive predictive value of 30%, and has a high rate of detection in healthy adults [6]. The imaging modality of choice is brain MRI which may reveal hyperintense T2-weighted abnormalities, especially in the temporal lobes; other areas that can be affected include the basal ganglia, diencephalon, brain stem, insular region, inferior frontal lobe, and cerebellar hemispheres [3]. CT is usually normal [3]. The use of electroencephalogram (EEG) in the diagnosis is controversial; there are some studies that have documented abnormalities in cases of acute and reactivated HHV-6 infection characterized by slow-wave activity in the temporal lobes which normalizes as the patient improves [2]. Brain biopsy is not routinely recommended [3].

There is a paucity of large, randomized, controlled trials to help guide treatment for patients with HHV-6 meningitis. Based on case reports, case series, and guidelines by the Infectious Diseases Society of America, ganciclovir or foscarnet are recommended as first-line therapy for immunocompromised patients; there are no studies looking at the effectiveness of these therapies in immunocompetent individuals [6]. There are case reports that suggest ganciclovir is effective and leads to complete remission [7]. Some patients may have lingering neurological symptoms despite antiviral therapy [4]. Cidofovir and brincidofovir are emerging as second-line agents [4]; however, these have not been approved for this indication yet [4]. Due to concerns over nephrotoxicity, ganciclovir is usually preferred over foscarnet or cidofovir [4]. Mutations in the phosphotransferase gene result in resistance to ganciclovir and cidofovir which sometimes complicates treatment [4]. Response to therapy is determined by improvement in symptoms and decreased viral load, if it was checked at the time of diagnosis [8]. Once antivirals are started, the yield from the viral load is low, and as demonstrated by this case, antiviral therapy sometimes leads to falsely low viral levels. The duration of treatment varies between three and four weeks or until the patient is asymptomatic. Treatment guidelines for HHV-6 encephalitis need to be further established.

## Conclusions

Meningitis is a rare presentation of HHV-6 infection in immunocompetent individuals. Given its low incidence, there are only a few case studies and case reports to help guide care when CNS involvement occurs. Minor neurological symptoms can be often overlooked (like early on in this case) which can lead to delays in diagnosis, treatment, and potentially increase the fatality of the disease. A high index of suspicion is required, especially after other more common causes have been ruled out. More studies regarding the HHV-6 and its neurological involvement are needed.
